# Metformin sensitizes leukemic cells to cytotoxic lymphocytes by increasing expression of intercellular adhesion molecule-1 (ICAM-1)

**DOI:** 10.1038/s41598-022-05470-x

**Published:** 2022-01-25

**Authors:** Nerea Allende-Vega, Joaquin Marco Brualla, Paolo Falvo, Catherine Alexia, Michael Constantinides, Alexis Fayd’herbe de Maudave, Lois Coenon, Delphine Gitenay, Giulia Mitola, Paul Massa, Stefania Orecchioni, Francesco Bertolini, Isabel Marzo, Alberto Anel, Martin Villalba

**Affiliations:** 1 IRMB, Univ Montpellier, INSERM, Montpellier, France; 2 Apoptosis, Immunity and Cancer Group, Department of Biochemistry and Molecular and Cell Biology, Faculty of Sciences, University of Zaragoza and Aragón Health Research Institute (IIS Aragón), Campus San Francisco Sq., 50009 Zaragoza, Spain; 3 Laboratory of Hematology-Oncology, European Institute of Oncology IRCCS, Milan, Italy; 4 CNRS, IRMB, INSERM, Univ Montpellier, CHU Montpellier, Montpellier, France; 5 Institut Sainte Catherine, Avignon, France

**Keywords:** Cancer, Immunology

## Abstract

Solid tumor cells have an altered metabolism that can protect them from cytotoxic lymphocytes. The anti-diabetic drug metformin modifies tumor cell metabolism and several clinical trials are testing its effectiveness for the treatment of solid cancers. The use of metformin in hematologic cancers has received much less attention, although allogeneic cytotoxic lymphocytes are very effective against these tumors. We show here that metformin induces expression of Natural Killer G2-D (NKG2D) ligands (NKG2DL) and intercellular adhesion molecule-1 (ICAM-1), a ligand of the lymphocyte function-associated antigen 1 (LFA-1). This leads to enhance sensitivity to cytotoxic lymphocytes. Overexpression of anti-apoptotic Bcl-2 family members decrease both metformin effects. The sensitization to activated cytotoxic lymphocytes is mainly mediated by the increase on ICAM-1 levels, which favors cytotoxic lymphocytes binding to tumor cells. Finally, metformin decreases the growth of human hematological tumor cells in xenograft models, mainly in presence of monoclonal antibodies that recognize tumor antigens. Our results suggest that metformin could improve cytotoxic lymphocyte-mediated therapy.

## Introduction

Tumor cells quickly proliferate and this requires a large amount of energy and biomass. To obtain them, tumors shift to a more glycolytic metabolism even in the presence of oxygen. This adaptation is generally known as the Warburg effect^1^ and is a therapeutic target for anticancer treatment^2^. Type 2 diabetic (T2D) patients show increase cancer incidence, but this is reverted in metformin-treated patients^3^. Metformin use in cancer, non-diabetic, patients is controversial and its pleiotropic effects are not fully understood^4^. The timing, dose and duration of treatment and the heterogeneity of the cancer patients enrolled in the different studies may contribute to different clinical benefits^4^. Moreover, the mechanism of action of metformin is complex. In vivo it selectively inhibits gluconeogenesis from redox-dependent substrates (lactate and glycerol), but not from redox-independent substrates (pyruvate, dihydroxyacetone phosphate (DHAP), alanine), which strongly indicates a redox-dependent mechanism of action^5^. In vitro and at higher doses, it inhibits mitochondrial complex I, fructose 1,6-bisphosphatase and c-AMP Response Element-binding protein (CREB) and activates AMP-activated protein kinase (AMPK;^5^).

In contrast to solid cancers, the use of metformin in hematologic cancers has received less attention^6,7^. Although certain positive clinical results have been described in some patients^8^, the results are in general poor or not revealed yet^6,7^. Metformin does not show clinical benefit in B chronic lymphocyte leukemia (B-CLL)^9^ or in acute myeloid leukemia (AML)^10^. Metformin is being tested in relapsed and/or refractory multiple myeloma (MM) patients in combination with Nelfinavir (NCT03829020), with high-dose of dexamethasone (NCT02967276) and with Ritonavir (NCT02948283). The results of these clinical trials are not available yet.

Immunotherapy is a promising approach to fight cancer. Changes in tumor metabolism impact interactions between immune and tumor cells^11–13^. Metformin can affect recognition of target cells by cytotoxic lymphocytes (CL;^4^) by modulating target metabolism. For example, metformin-induced activation of AMPK promotes PD-L1 phosphorylation, resulting in endoplasmic reticulum-associated PD-L1 protein degradation, which increases cytotoxic T-lymphocyte (CTL)-mediated tumor cell death^14^. Metformin-induced metabolic changes can also stress tumor cells and increase expression of the stress-induced ligand MHC Class I Polypeptide-Related Sequence A (MICA)^15^. MICA, and other stress-induced ligands such as MICB and UL16 Binding Proteins (ULBPs), are recognized by activating receptors in CL, e.g. the Natural Killer G2-D (NKG2D), leading to NK cell activation. These stress-induced ligands are also called NKG2D ligands (NKG2DL)^16^.

Metformin modulates the activity of p53, a master regulator of cell metabolism, via AMPK activation in several cancer contexts^17–19^, including hematological cancer cells^20^. Human *ULBP1* and *2* genes contain consensus p53 response elements, and p53 probably amplifies transcription of certain human *NKG2DL* mRNAs^16,21^. Therefore, p53 is a possible candidate to link metabolism and expression of NKG2DL.

Cancer and aging patients have impaired NK function^22^. This could decrease the clinical activity of antitumor drugs designed to recruit NK cells. In this sense the lack of clinical activity of metformin in certain cancer patients could be related to the advanced patient’s age at treatment^10,23^. Therefore, the use of allogeneic NK cells together with metformin could improve clinical treatment. We have developed a protocol to activate and expand umbilical cord blood (UCB)-derived NK cells in vitro^24^. These expanded NK (eNK) have high cytolytic activity against hematological cancer cells, even those resistant to chemotherapy^24–26^. We have investigated here if metformin sensitizes hematopoietic cells to CL and the possible mechanism(s) of action.

## Results

### Metformin regulates expression of stress ligands and ICAM-1 in leukemic cells

**Figure 1:**
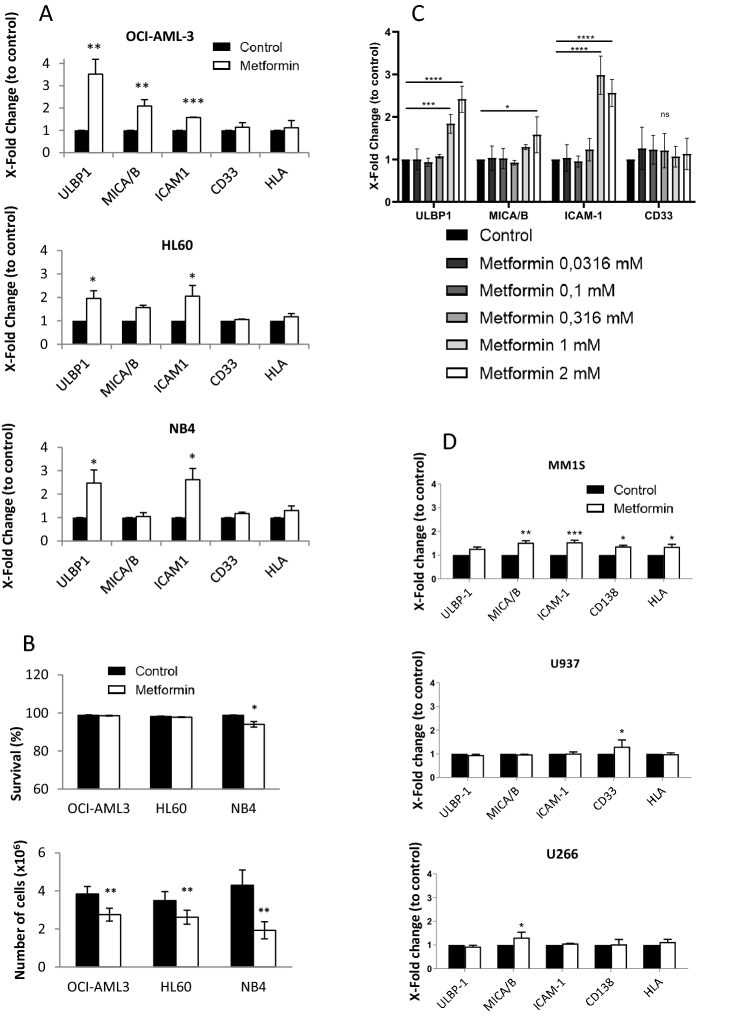
Metformin regulated expression of stress ligands and ICAM-I in leukemic cells. (**A**) Different AML cell lines OCI-AML-3 wtp53, HL-60 nullp53, NB4 mutp53 were treated with 2 mM metformin for 3 days and plasma membrane expression of the stress ligands MICA/B and ULBP1, the integrin ICAM-1, the myeloid marker CD33 and MHC class I HLA were analyzed by FACs analysis. (**B**) The different cell lines were treated as in (**A**) and the total number of cells and the percentage of alive cells were measured on the Muse Cell Analyzer. (**C**) OCI-AML3 cells were treated with different concentrations (0.0316; 0.1; 0.316; 1 and 2 mM) of metformin for 3 days, metformin was added every day and plasma membrane expression of the stress ligands MICA/B and ULBP1, the integrin ICAM-1 and the cell line marker CD33 were analyzed by FACs analysis. (**D**) Different MM cell lines MM1.S wtp53 and U266 mutp53 and the pro-monocytic myeloid leukemia cell U937 nullp53 were treated with 2 mM metformin for 3 days and the expression of the above described markers plus the MM cell line marker CD138 were analyzed by FACs. Data represent the Mean Fluorescence Intensity (MFI) levels compared to control, non-treated, cells. The bar graphs represent means ± SD of 3 independent experiments; *p < 0.05, **p < 0.01, ***p < 0.005 student t-test compare to control cells.

We have previously shown in several studies that the presence of wtp53 impacts the effect of several co-treatments involving the so-called metabolic drugs, e.g. metformin^20,27,28^. Hence, we investigated if p53 status can affect the effect of metformin on ligands that are recognized by cytotoxic lymphocytes. We treated with 2 mM metformin for 3 days 3 acute myeloid leukemia (AML) cell lines with different p53 status (OCI-AML3 cells express wt p53, HL60 are p53 null and NB4 express mutant (mut) p53^20,29^) and analyzed MICA/B and ULBP1 expression on plasma membrane. In addition, we evaluated levels of other ligands such as intercellular adhesion molecule-1 (ICAM-1), a ligand of the lymphocyte function-associated antigen 1 (LFA-1). ICAM-1/LFA-1 interaction is essential for target cell recognition by CL^30,31^. We studied also MHC-I, called HLA in humans, which is the ligand of the killer-cell immunoglobulin-like receptors (KIRs), the main NK cell inhibitory receptors^22^ and the myeloid marker CD33. Metformin did not statistically change expression of the last two molecules in any cell line (Fig. 1A and Supplemental Fig. 1). In contrast, it increased expression of ULBP-1 and of the integrin ligand ICAM-1 in all of them and of MICA/B in OCI-AML3 cells (Fig. 1A and Supplemental Fig. 1). Of note, metformin was cytostatic, but not cytotoxic, on these leukemic cells at the selected dose (Fig. 1B). We next tested lower doses of metformin in the cell line that better respond to treatment, i.e. OCI-AML3 (Fig. 1C). Whereas 1 and 2 mM of metformin gave similar effects, concentrations under 1 mM lacked effect. Therefore, for future experiments we used 2 mM.

We challenged these results in another set of hematological cancer cells: the multiple myeloma (MM) cell lines MM1.S (wtp53) and U266 (mtp53) and the pro-monocytic myeloid leukemia cell U937 (nullp53)^20,29,32^. We treated them with metformin and analyzed the previously described antigens, but using the MM marker CD138 instead of CD33 for the MM cell lines. Metformin increased all markers in MM1.S cells, but only MICA/B in U266 and CD33 in U937 cells (Fig. 1D). Hence, in both cell line cohorts, we observed a higher response to metformin in cells expressing wtp53.

### Overexpression of antiapoptotic Bcl-2 family members affects metformin effects on leukemic cells

**Figure 2:**
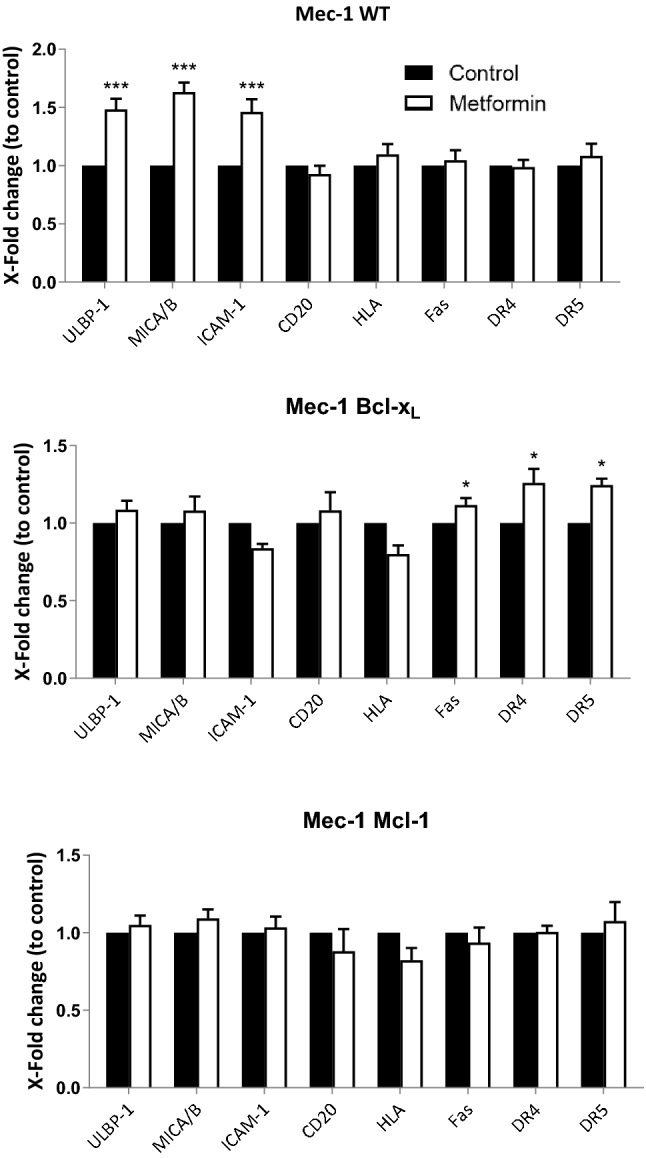
Overexpression of antiapoptotic Bcl-2 members affected metformin effects on leukemic cells. (**A**) Mec-1 cell line and stable cell lines overexpressing Bcl-X_L_ or Mcl-1 were incubated with 2 mM metformin for 3 days and plasma membrane expression of the stress ligands MICA/B and ULBP1, the integrin ICAM-1, the death receptors Fas, DR4 and DR5, the (**B**) cell marker CD20 and MHC class I HLA were analyzed by FACs analysis. Data represent the amount of protein compared to control, non-treated, cells. The bar graphs represent means ± SD of 3 independent experiments; *p < 0.05, **p < 0.01, ***p < 0.005 student t-test compare to control cells.

We next investigated the effect of metformin on cells that display chemoresistant mechanisms. Bcl-X_L_ overexpression is associated with bad prognosis and chemoresistance in several types of tumors^33,34^. Mcl-1 overexpression is linked to chemoresistance in multiple myeloma^35,36^. Both proteins are usually overexpressed in tumor cells from chemoresistant patients. Figure 2 and supplemental Fig. 2 showed that metformin induced expression of ICAM-1, ULBP1 and MICA/B on the B-cell chronic lymphocytic leukemia (B-CLL) cell line MEC1 (mutp53). But, it did not increase expression of HLA, the B cell marker CD20 or the death receptors Fas, DR4 or DR5. In cells overexpressing Bcl-X_L_, metformin only increased expression of the above-mentioned death receptors. Metformin failed to change expression of any of these antigens in cells overexpressing Mcl-1.

### Metformin sensitizes leukemic cells to cytotoxic lymphocytes (CL)

**Figure 3:**
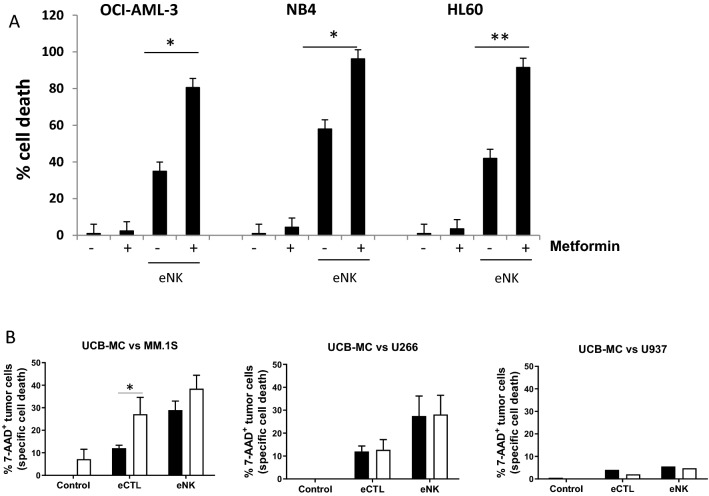
Metformin sensitizes leukemic cells to NK cells. (**A**) OCI-AML3, NB4 and HL60 AML cell lines were treated with 2 mM metformin for 3 days before overnight incubation at 1:1 effector:target (E:T) ratio with e-NK cells. (**B**) Different MM cell lines were treated with 2 mM metformin (white bars) for 3 days before overnight incubation at 1:1 E:T ratio with e-NK cells or eCTLs. The specific killing of tumor cells (7-AAD^+^) was quantified by FACs. UCB-MC stays for Umbilical Cord Blood—Mononuclear Cells. The data represent means ± SD; *p < 0.05, **p < 0.01, Student's t-test compared to control cells or as depicted in the graphic.

We next analyzed metformin effect on CL-mediated tumor cell killing. We generated expanded and activated NK cells (eNK) from umbilical cord blood (UCB) as previously described^24–26^ and used them against the AML cell lines described in Fig. 1A. As described in Fig. 1, metformin barely affected cell survival but eNK killed between 30 and 60% of target cells, depending on the cell line (Fig. 3A and supplemental Fig. 3). Target cell pretreatment with metformin doubled eNK-mediated killing in all cell lines. Similarly, in the cell lines described in Fig. 1C, metformin increased killing of MM1.S cells, but not the other cell lines lacking wtp53 (Fig. 3B). We generated expanded cytotoxic T lymphocytes (eCTL) as previously described^37^. Metformin sensitized MM1.S cells to them, but not U266 or U937 (Fig. 3B). This is in agreement with our previous results showing that some of the metformin effects in these leukemic cells depend on p53 status^20^.

### Overexpression of anti-apoptotic Bcl-2 members blocks metformin-induced sensitization of leukemic cells

**Figure 4:**
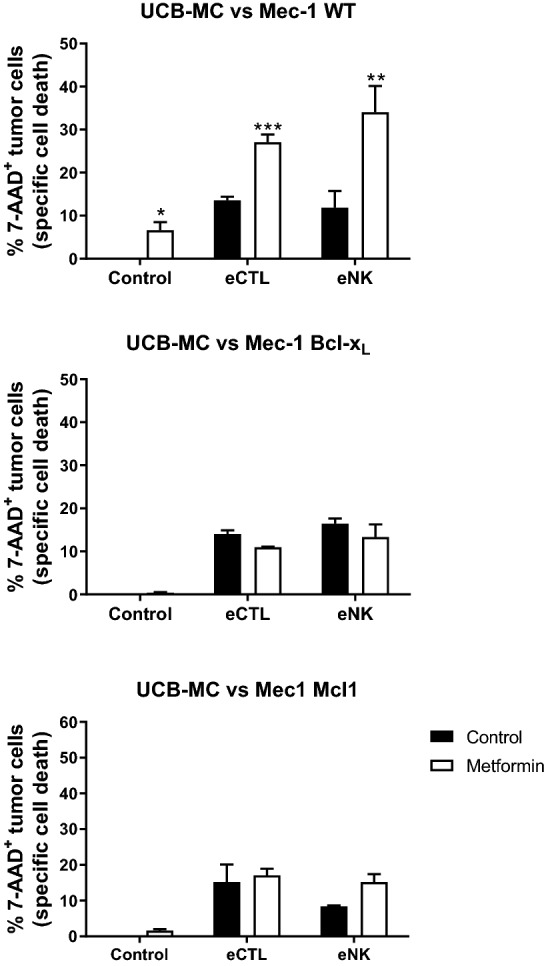
Overexpression of antiapoptotic Bcl-2 members blocks metformin-induced sensitization of leukemic cells. Mec-1 cell line and stable cell lines overexpressing Bcl-X_L_ or Mcl-1 were incubated with 2 mM metformin (white bars) for 3 days before overnight incubation at 1:1 E:T ratio with e-NK cells or eCTLs. The specific killing of tumor cells (7-AAD^+^) was quantified by FACs. The data represent means ± SD; *p < 0.05, **p < 0.01, ***p < 0.001 Student's t-test compared to cells not treated with metformin.

We treated with metformin the cell lines described in Fig. 2 and then tested them to eNK and eCTL cytotoxicity. Metformin clearly sensitized Mec1wt cells to both eNK and eCTL cytotoxicity while it did not have any significant effect on cells overexpressing Bcl-x_L_ or Mcl-1, although they were somewhat sensitive to their cytotoxicity, as previously described^24^ (Fig. 4).

### Metformin sensitizes primary leukemic cells to NK cells

**Figure 5:**
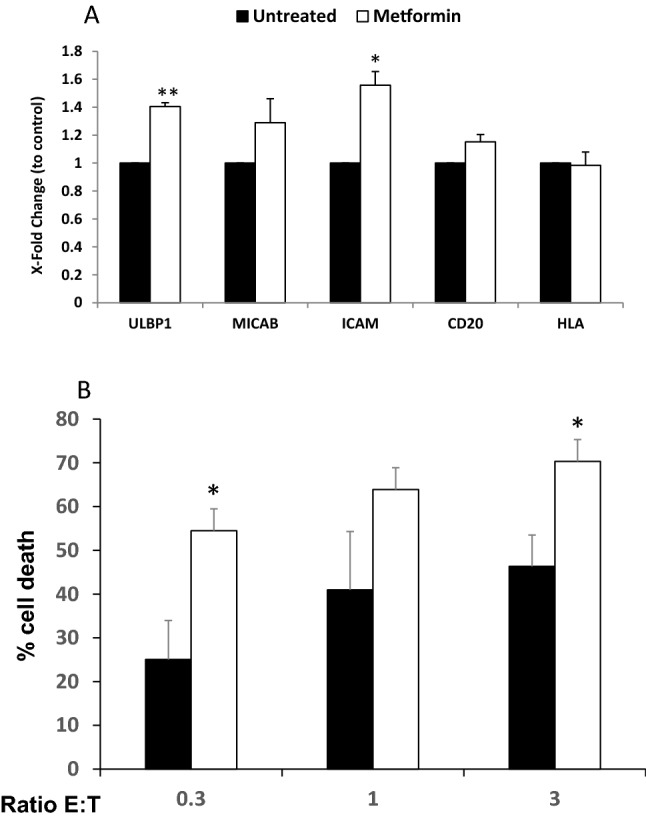
Metformin sensitizes primary leukemic cells to NK cells. (**A**) BCL-P2 cells were treated with 2 mM metformin for 3 days and plasma membrane expression of the stress ligands MICA/B and ULBP1, the integrin ICAM-1, the B cell marker CD20 and MHC class I (HLA) were analyzed by FACs. (**B**) BCL-P2 cells were treated with metformin as in (**A**) before overnight incubation at different E:T ratios with e-NK cells derived from 4 donors. The specific death of tumor cells (7-AAD^+^) was quantified by FACs. The data represent means ± SD; *p < 0.05, **p < 0.01, ***p < 0.001 Student's t-test compared to control cells or as depicted in the graphic.

We then treated with metformin the primary cells from a B-cell lymphoma patient (BCL-P2) and observed that it significantly increased expression of ULBP1 and ICAM-1 and tended to increase MICA/B and CD20 expression (Fig. 5A and Supplemental Fig. 4). In contrast, we did not find a significant effect on HLA surface expression. eNK cells killed BCL-P2 cells and metformin significantly increased killing (Fig. 5B and Supplemental Fig. 5).

#### Metformin mainly favors leukemic cell killing through an increase in LFA-1/ICAM-1 interaction

**Figure 6:**
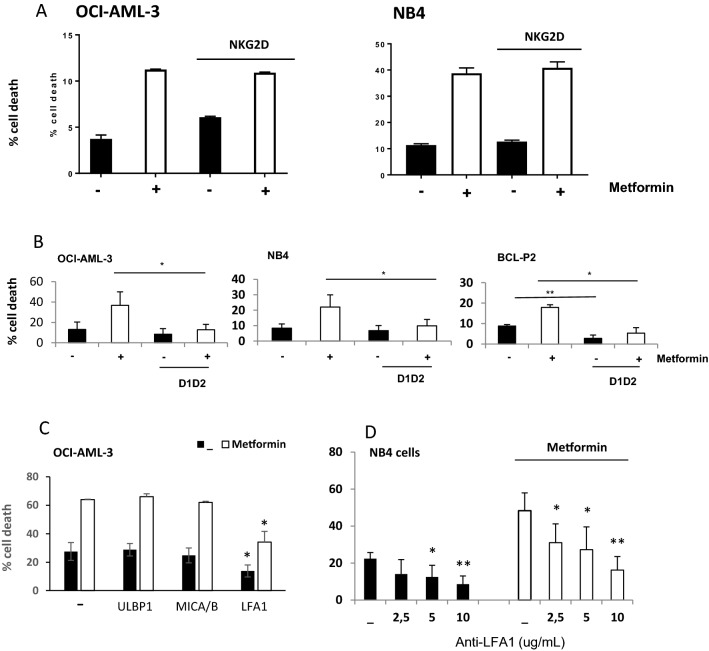
Metformin mainly favors leukemic cell killing through an increase in LFA-1/ICAM-1 interaction. (**A**) OCI-AML-3 or NB4 cells were treated with 2 mM metformin for 3 days and later incubated with 20 µg/ml of an antibody (MAB139) blocking NKG2D activity before overnight incubation at 1:1 E:T ratio with e-NK cells. (**B**) The different cell lines were treated as in (**A**) but they were incubated with 15 µg/ml of a LFA-1 blocking construct. (**C**) OCI-AML-3 cells were incubated as in (**A**) but in the presence of anti-ULBP1 (10 µg/ml), anti-MICA/B (10 µg/ml) or anti-β2 integrin (2, 5–10 µg/ml) blocking antibodies. (**D**) NB4 cells were incubated with different concentrations of ICAM-1 blocking peptides. The specific killing of tumor cells (7-AAD^+^) was quantified by FACs. The data represent means ± SD; *p < 0.05, **p < 0.01, ***p < 0.001 Student's t-test compared to control cells or as depicted in the graphic.

The increase in the expression of stress ligands suggested that metformin-induced sensitization of tumor cells could rely on the interaction of NKG2D with its ligands. Hence, we incubated eNK cells with an anti-NKG2D mAb that blocks the binding of NKG2D with NG2DL before putting them in contact with OCI-AML3 and NB4 cells (Fig. 6A). Although our eNK express NKG2D levels similar to non-activated NK cells^25^, we did not observe any effect on cell killing in control or metformin-treated cells. In contrast, when we used a construct (D1D2) that binds to LFA-1 and blocks its binding to ICAM-1^24^, we observed a significant reduction in cell killing in metformin-treated cells in three leukemic cell types, including primary BCL-P2 cells (Fig. 6B).

We next used another set of antibodies to block the interaction of these receptors with their ligands (Fig. 6C). Blocking ULBP1or MICA/B with specific antibodies did not affect eNK killing. In contrast, when we used an antibody against integrin β2, a component of LFA-1, we observed a significant reduction in cell death in both, control and metformin-treated cells. The strong requirement for LFA1/ICAM1 interaction was also observed in NB4 cells (Fig. 6D). In fact, the decrease on tumor cell recognition by blocking ICAM-1/LFA-1 interaction was strongly dependent on the concentration used.

### Metformin delays growth of a fast-growing lymphoma in vivo in the presence of an anti-CD20 mAb

**Figure 7:**
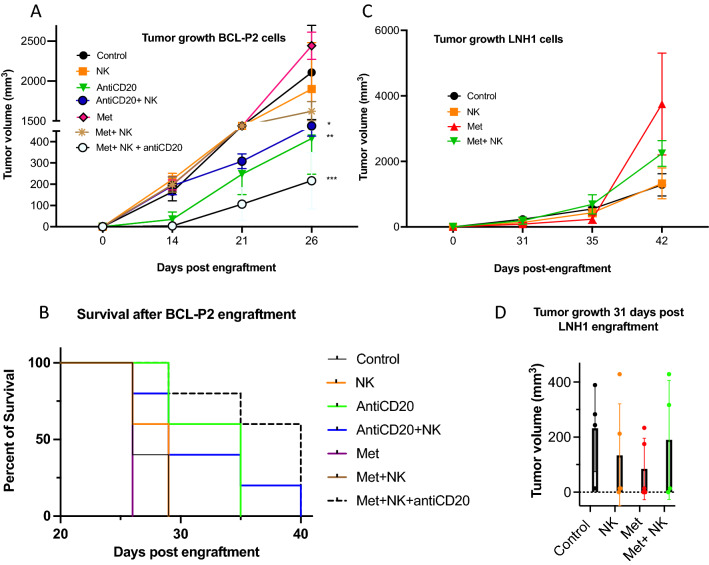
Metformin slightly affect cell tumor growth in NSG mice engrafted with lymphoma cells. (**A**) Five NSG mice/group were subcutaneously engrafted with 5 × 10^6^ BCLP2 and treated with metformin, with e-NK and/or with an anti-CD20 mAb. Graphs show tumor cell growth. (**B**) Mice survival after different treatments. (**C**) Five NSG mice/group were subcutaneously engrafted with 10 × 10^6^ LNH1 (right) cells and treated with metformin and/or with e-NK. Graphs show tumor cell growth. (**D**) Tumor cell growth at day 31 after LNH1 engraftment. Graphs show means ± SD, *p < 0.05, **p < 0.01, ***p < 0.005; 2way Anova compared to non-treated cells.

We next investigated the effect of metformin on lymphoma cell growth in vivo*.* We used BCL-P2 cells that quickly induce tumors when engrafted subcutaneously at 5 million cells per mice in immunosuppressed NSG mice^24^. Metformin alone did not affect tumor development while adoptive transfer of eNK cells barely affected it (Fig. 7A). Consequently, mice survival was not affected by these treatments (Fig. 7B). Tumor growth was delayed by the anti-CD20 mAb rituximab which also increased mice survival (Fig. 7). eNK cell combination with this effective anti-CD20 mAb did not statistically further decrease tumor growth or mice survival (Fig. 7). In contrast, the combinatory treatment of metformin, eNK and anti-CD20 mAb significantly reduced tumor growth with respect to the double combinatory treatments (Fig. 7A), i.e. metformin + eNK and antiCD20 + eNK, and also increased mice survival (Fig. 7B).

### Metformin tends to delay growth of a slow-growing lymphoma in vivo

We next investigated metformin effects on the growth of LNH1 cells, which are issued from a diffuse large B-cell lymphoma (DLBCL) patient. These cells grow slower than BCL-P2 when subcutaneously engrafted at 10 million cells per mice^24^. In these experimental conditions eNK cells alone failed to affect tumor growth (Fig. 7C). However, metformin treatment tends to delay tumor development although combination with eNK cells did not have any additional effect (Fig. 7D). The anti-CD20 mAb totally abrogated the growth of these cells (data not shown), making irrelevant the cotreatment with Metformin and/or eNK.

## Discussion

Metformin has an undoubtedly anticancer value^4,5^, but its mechanism of action is complex and not fully understood. Its immunomodulatory effect should be taken into account to optimize its clinical use^4^. This could be more relevant on hematological cancers in which effector immune cells are in close contact to malignant cells because they niche in the same tissues. Several clinical approaches such as CAR T^38^ or CAR NK^39^ cells have shown a relevant success to treat blood-borne cancers. However, roughly 50% of patients undergo relapse and new clinical protocols are required to improve the clinical activity of these genetically-modified cytotoxic lymphocytes. Expression of the PD-1 immune checkpoint ligand PD-L1 in target cells efficiently blocks CTL killing. Metformin can promote PD-L1 phosphorylation and degradation^14^. This could increase cytotoxic lymphocyte-mediated tumor cell death. The PD-1/PD-L1 immune checkpoint is less relevant in NK-mediated killing and, additionally, we have observed that our eNK cells express low levels of PD-1^26^. Moreover, in MM samples pembrolizumab does not increase tumor killing by our eNK^26^. Hence, we do not believe that this immune checkpoint plays a main role in our observations.

Initially, we believed that metformin sensitizes leukemic cells to NK by induction of stress molecules in target cells. We certainly reproduce previous findings^15^ showing that metformin increases expression of stress ligands in multiple cell lines. Our eNK express similar NKG2D levels to naïve cells^25^, but blocking NKG2D or MICA/B and ULBP1 does not decrease metformin effect. However, we have used activated NK cells. Hence, it is possible that naïve NK cells can better sense NKG2DL upregulation on tumor targets. If naïve or activated cells are more representative of the situation in the tumor microenvironment is unclear. It is believed that once in the tumor microenvironment NK cells should be activated by the targets and/or the pro-inflammatory cytokines. In contrast, for tumor cells outside of this environment, e.g. metastatic cells, it could be the opposite. Moreover, in patients the effect of metformin can be different than in our mouse models. In summary, we cannot exclude a major role of metformin in patients.

The clinical results regarding the use of metformin in cancer treatment are inconclusive^4^. In fact, the timing, dose and duration of treatment and the heterogeneity of the patients enrolled make difficult to compare the different trials^4^. In vivo metformin inhibits gluconeogenesis from redox-dependent substrates^5^. In vitro and at higher doses, it increases expression of MICA^15^. But, most importantly, metformin inhibits mitochondrial complex I, fructose 1,6-bisphosphatase and CREB and activates AMPK;^5^. All these effects can target multiple downstream signaling pathways and the most studied is AMPK downstream signaling^5^. AMPK promotes PD-L1 protein degradation^14^, activates p53^17–20^, inhibits lipid synthesis^40^ and regulates mTORC1 axis^41^. The role of each of these pathways in ICAM-1 and/or NKG2DL expression is unknown. It is also largely possible that several pathways play different roles, finally leading to expression of those CL ligands and tumor sensitization to CLs. It would be extremely interesting to fully unveil this pathway to better design new immunotherapies to improve the clinical activity of allogeneic CLs^11,12^.

Interestingly, the cell lines U266 and U937, which are not sensitized to cytotoxic lymphocytes by metformin, do not upregulate ICAM-1 after metformin treatment. Moreover, Mec-1 cells overexpressing the Bcl-2 family members Bcl-X_L_ or Mcl-1 also failed to upregulate ICAM-1 and are resistant to metformin-induced sensitization. Finally, blocking ICAM-1/LFA-1 interaction strongly decreases metformin effect. Hence, our results support that metformin mediates upregulation of integrin ligands, i.e. ICAM-1, which allows the effective binding and activity of cytotoxic lymphocytes on tumor cells^42^. Cytotoxic lymphocytes travel and roll over multiple substrates and sample different cellular environments. They need to be able to stop, process the stimulating signals of transient cellular contacts and move on^42^. However, when these stimulatory signals are strong enough they should stop, form an adherent junction, stable and strong with the target cell and eliminate it. Metformin-induced ICAM-1 expression on target cells should favor that NK cells establish close contact with them. If the stimulating signals are strong enough, NK cells will proceed to kill the target^30,31^. This has been described in in vitro activated NK cells using a similar expansion protocol as the one we have used here^31^. Interestingly, metformin decreases ICAM-1 expression in non-transformed cells^43–45^, including polycystic ovary syndrome (PCOS) subjects^46^ and patients with T2D^47^. Hence, it should not favor cytotoxic lymphocyte binding to healthy tissue that could induce autoimmunity.

We did not find any effect in our system. However, we used activated NK cells which are those that should infiltrate the tumor. Hence, it is possible that naïve NK cells can better sense NKG2DL upregulation. Moreover, in patients the effect can be different than in mouse models. We have included these comments in the discussion section.

Metformin only slightly delays tumor growth and does not improve mice survival either alone or together with eNK. In contrast, it improves survival and decreases tumor growth in the presence of an anti-CD20 mAb and eNK. In our mouse model, eNK are in contact with metformin during their antitumor function, which is not the case of the in vitro experiments. It is well-known that cytotoxic lymphocytes require a glycolytic metabolism for their maximal activity and educated NK cells display a high glycolytic rate^48^, which is essential for their antitumor function^49^. Hence, metformin could affect NK cell metabolism in vivo impairing their function. The efficient anti-CD20 mAb could overpass this effect by inducing maximal NK cell activation through the legation of CD16, which recognizes the Fc moiety of Abs. In this context, metformin improves outcome of DLBCL patients at least partially by sensitizing cells to the anti-CD20 rituximab^50^. However, we do not know if our results apply to naïve NK cells. eNK have a relatively high basal cytotoxicity compared to naïve NK cells and show a mature phenotype^24–26^. The more mature NK subsets, which possess higher cytotoxic potential, show the highest activation by LFA-1^30^. Therefore, it is possible that metformin-induced NK sensitization is specific of already activated cytotoxic lymphocytes, something that perhaps is uncommon in cancer patients that carry impaired NK activity^22^.

The concentration of metformin in plasma in T2D patients is around 30 µm^51^. A daily dose of metformin could reach 2,000 mg a day^4^, which is basically 15 mmol and could perhaps locally give higher metformin concentrations. For example, it can reach 140 µm in liver^4^. The intratumor concentration of metformin is difficult to evaluate, but it accumulates in ovarian cancer patient biopsies^52^. Moreover, metformin concentration reaches on average 0.41 mmol/kg in the colon of colorectal cancer patients daily treated with an oral low dose of 250 mg/d of metformin, with some patients reaching 1.87 mmol/kg^53,54^. In addition, and remarkably, metformin effects are higher at low glucose concentration (1 mM), which are found in tumor microenvironment^52^. This is probably the case of AML and MM patient bone marrow, which is highly infiltrated by glucose-consuming tumor cells. Hence, metformin concentration in the tumor can be significantly higher than in plasma and/or it can produce specific effects at lower doses in the tumor microenvironment.

The antitumor function of metformin is very heterogenous. As previously described, the large variety on age and type of disease of the patients engaged in the clinical trials could explain this fact. We show here that p53 status and/or overexpression of Bcl-x_L_ or Mcl-1 could make tumor cells resistant to the metformin-induced sensitization to cytotoxic lymphocytes. Moreover, the activity of NK cells is largely impaired in leukemia patients^22^ and our results suggest that metformin could give better clinical results in patients with sufficient NK activity. Hence, we believe that metformin together with allogeneic activated NK cells could be a future relevant treatment. The ongoing clinical studies of metformin in nondiabetic, cancer, patients will soon show the effect of metformin in several clinical contexts and perhaps support the use of such co-treatment.

## Materials and methods

### Ethical statement

Experiments involving animals were approved by the Italian National Institute of Health the 08/29/2019 under the number 639/2019 and have been done in accordance with the applicable Italian laws (D.L.vo 26/14 and following amendments), with the Institutional Animal Care and Use Committee, and with the institutional guidelines at the European Institute of Oncology.

### Cell lines and culture conditions

The AML cell lines OCI-AML3, NB4 and HL-60 and the MM cell lines MM1.S, U266 and U937 were grown in RPMI 1640–Glutamax (GIBCO) supplemented with 10% FBS^20,28^. Primary cells from a lymphoma B cell patient (BCL-P2) were grown in the same medium with 10% FBS.

### Reagents and antibodies

Metformin was from Santa Cruz Technologies. Fluorescence coupled antibodies against human ICAM-1 (AF1730), ULBP-1 (MAB1380), MICA/B (MAB13001) and the blocking antibody against NKG2D (MAB139) were from R&D Systems; CD33, HLA and CD138 were from Beckman; DR4 (12-6644-73), DR5 (12-9908-42) and Fas (BMS140FI) were from eBiosciences and 7AAD from BD Biosciences. For in vivo experiments we used the anti-CD20 rituximab. The D1D2 construct that binds to LFA-1 has been described^24^. Blocking anti-ULBP1 antibody MAB1380, anti-MICA/B antibody MAB13001 and anti-β2 integrin AF1730 were from R&D systems.

### NK cell isolation and expansion

This work benefited from umbilical cord blood units (UCBs) and the expertise of Prof. John De Vos, in charge of the Biological Resource Center Collection of the University Hospital of Montpellier—http://www.chu-montpellier.fr/en/platforms (BIOBANQUES Identifier—BB-0033-00031). NK cells were expanded as previously described^24,26^. Briefly, UCBMCs or PBMCs were isolated through density gradient centrifugation using Histopaque-1077 (Sigma). Blood samples were diluted at 1:1 ratio with RPMI then layered above 10 mL Histopaque in a 50 mL conical tube. Once centrifuged for 30 min at 400×*g*, the white layers of mononuclear cells (MCs) were collected and washed. Using EasySep Human CD3 Positive Isolation kit (StemCell Technologies), the CD3^+^ cell fraction (T and NKT cells) of the MCs was depleted in each sample to better culture the NK cells. Once depletion was verified through flow cytometry, cells were cultured for 20 days. NKs were cultured with γ-irradiated PLH at a 1:4 (NK cell: feeder cell) ratio and IL-2 (100 IU/mL) and IL-15 (5 ng/mL). Feeder cells and cytokines were refreshed every 3–4 days. To monitor expansion, NK cells were stained with APC-labelled anti-CD3 mAb and PE or Vio770-labelled anti-CD56 mAb (both form BD Biosciences). Culture viability was determined at regular intervals through flow cytometry analysis.

### eCTL expansion

All eCTLs used were generated from PBMCs of healthy donors obtained from leukopaks provided by the Blood and Tissue Bank of Aragón, under the permission of the Clinical Research Ethical Committee from Aragón (CEICA) (Ref. PI16/0129). Briefly, PBMCs were isolated through density gradient centrifugation using Histopaque-1077 (Sigma), as stated above. Afterwards, PBMCs were cultured for 20 days with PLH (previously inactivated with mitomycin C at 25 µg/mL) at a 1:1 PBMC:feeder cell ratio plus IL-2 (100 IU/mL) and IL-15 (5 ng/mL). Feeder cells and cytokines were refreshed every 3–4 days. Expansion was monitored by PBMC staining with mAb CD3-FITC or mAb CD56-APC, both from Miltenyi Biotech. Culture viability was determined at regular intervals through flow cytometry analysis. Once obtained eCTL, CD3^+^ population was selected using EasySep Human CD3 Positive Isolation kit (StemCell Technologies) and CD3^+^/CD56^-^ presence was confirmed by flow cytometry.

### In vivo experiments

In vivo experiments were carried out using 6–8 weeks/old male NOD scid gamma (NSG) mice, which were bred and housed in pathogen-free conditions in the animal facility of the European Institute of Oncology–Italian Foundation for Cancer Research (FIRC), Institute of Molecular Oncology (Milan, Italy). For engraftment of human cells, mice were subcutaneously engrafted with 5 × 10^6^ BCL-P2 or 10 × 10^6^ LNH1 primary tumor cells derived from a BCL (P2) patient or a diffuse large B-cell lymphoma (DLBCL) patient (LNH1). Metformin was given ad libitum through the drinking water at 2 mg/ml. At day 4, we engrafted 10 (BCL-P2) or 10 (LNH1) million e-NK cells and at day 6, mice were treated i.p. with the anti-CD20 rituximab (in saline medium) 3 mg/kg once a week × 3 weeks; or with a combination of both. Tumor growth was monitored at least once a week using a digital calliper, and tumor volume was calculated according to the formula: L × W^2^/2 = mm^3^, where W represents the width and L the length of the tumor mass.

### Counting and determination of cell viability

After treatment, hematopoietic cells were stained with Muse Count and Viability Reagent, and then analyzed on the Muse Cell Analyzer (Millipore) to identify cell number and survival^28^.

### NK cell mediated cytotoxicity

Target cells were incubated with 2 mm Metformin for 3 days. After washing, cells were suspended in RPMI medium 10% FBS and 100.00 cells per well (96 well round (U) bottom plate) were incubated overnight in the presence or absence of e-NK cells at a 1: 1 effector: target (E: T) ratio. Previously fresh or frozen (stored in liquid nitrogen) NK cells were labeled with 3 µm of CellTracker™ Violet BMQC Dye (Life Technologies). Subsequently, viability was analyzed in the violet fluorescence negative target cell population by flow cytometry (Galllios Beckman Coulter) using 7AAD (BD Biosciencies)^24^.

### Flow Cytometry

Briefly, 1 × 10^6^ cells were stained with antibody in PBS with 2% FBS and incubated at 37 °C for 30 min. Cells were then washed and suspended in 200–250 μl PBS 2% FBS with the corresponding antibodies. Staining was analyzed using a Gallios flow cytometer (Beckman) and Kaluza software.

### Statistical analysis

The statistical analysis of the difference between means was performed using the 2way ANOVA test or the Student’t test using the software Prism9 from GraphPad Software, LLC. The results are given as the confidence interval (*:*p* < 0.05, **:*p* < 0.01, ***:*p* < 0.005). All the experiments described in the figures with a quantitative analysis have been performed at least three times in duplicate. Other experiments were performed three times with similar results.

## Supplementary Information

Supplementary Figures.
