# Supplementary material for: Metformin sensitizes leukemic cells to cytotoxic lymphocytes by increasing expression of intercellular adhesion molecule-1 (ICAM-1)
Source: Sci Rep. 2022 Jan 25;12:1341. doi: 10.1038/s41598-022-05470-x (PMC8789909; doi:10.1038/s41598-022-05470-x)
Supplement: Supplementary file 1 — Supplementary Figures. [file 41598_2022_5470_MOESM1_ESM.pptx]

## Slide 1
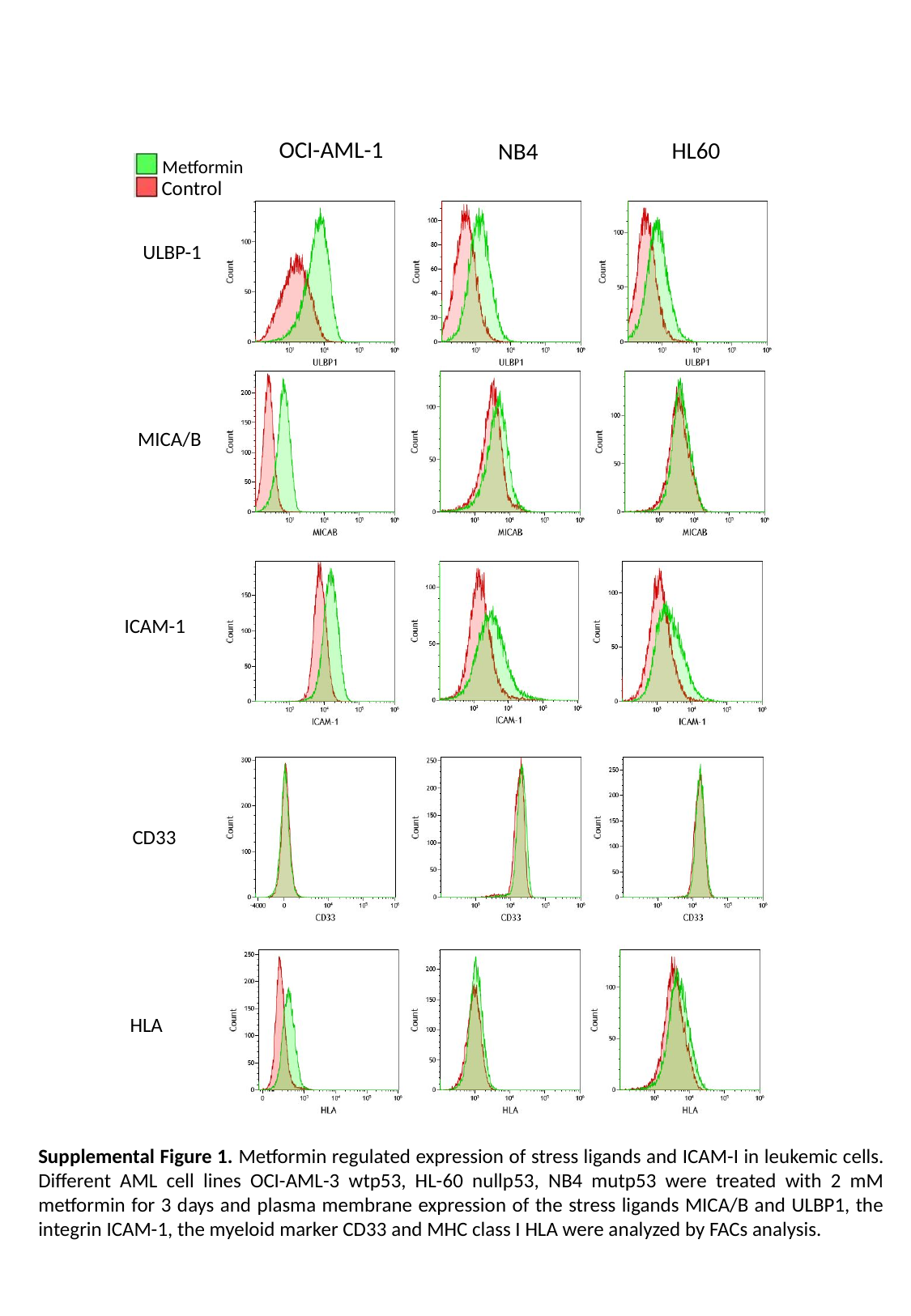

OCI-AML-1
HL60
NB4
ULBP-1
MICA/B
ICAM-1
CD33
HLA
Metformin
Control
Supplemental Figure 1. Metformin regulated expression of stress ligands and ICAM-I in leukemic cells. Different AML cell lines OCI-AML-3 wtp53, HL-60 nullp53, NB4 mutp53 were treated with 2 mM metformin for 3 days and plasma membrane expression of the stress ligands MICA/B and ULBP1, the integrin ICAM-1, the myeloid marker CD33 and MHC class I HLA were analyzed by FACs analysis.

## Slide 2
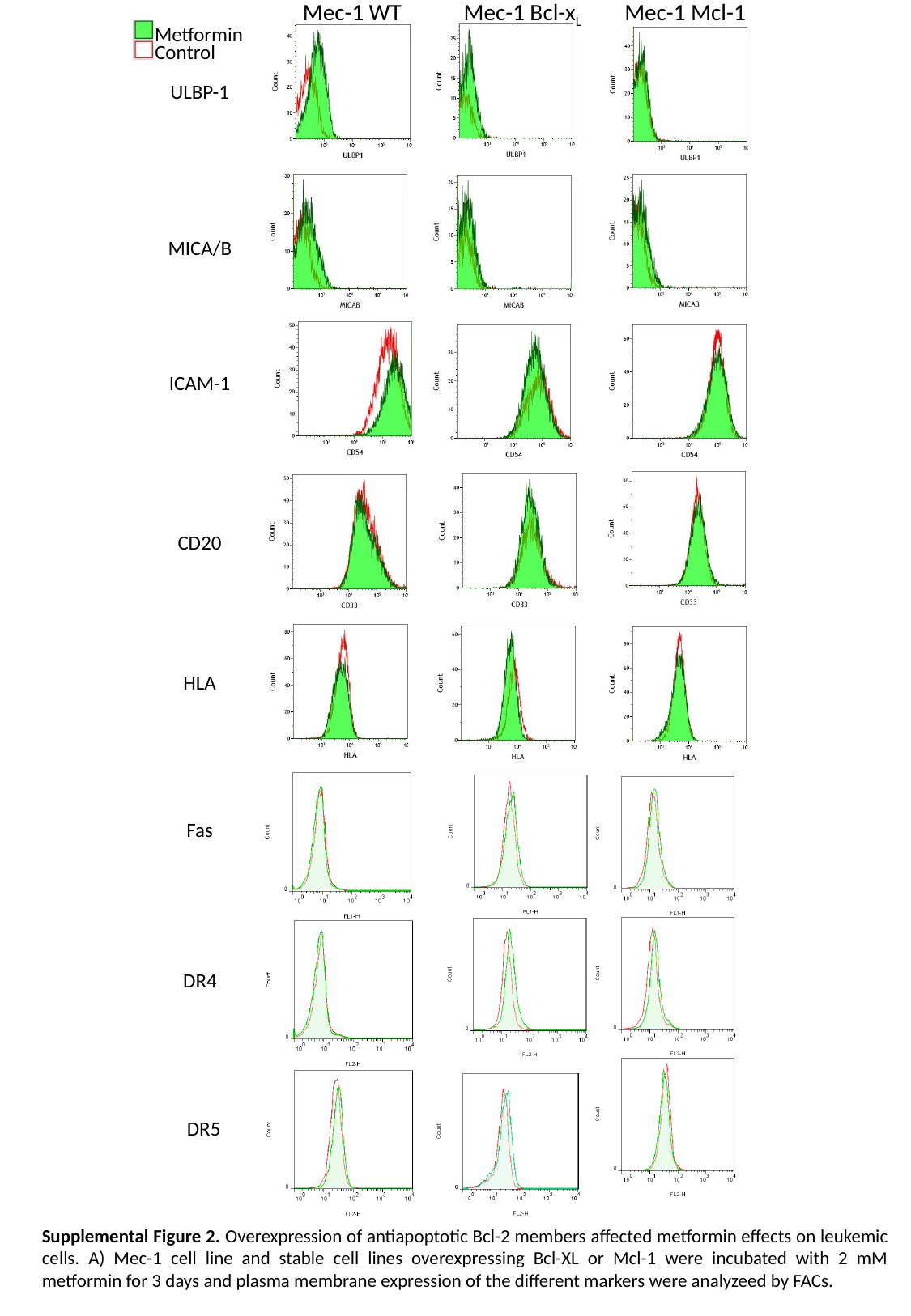

Mec-1 Bcl-xL
Mec-1 WT
Mec-1 Mcl-1
Metformin
Control
ULBP-1
MICA/B
ICAM-1
CD20
CD20
CD20
CD20
HLA
Fas
DR4
DR5
Supplemental Figure 2. Overexpression of antiapoptotic Bcl-2 members affected metformin effects on leukemic cells. A) Mec-1 cell line and stable cell lines overexpressing Bcl-XL or Mcl-1 were incubated with 2 mM metformin for 3 days and plasma membrane expression of the different markers were analyzeed by FACs.

## Slide 3
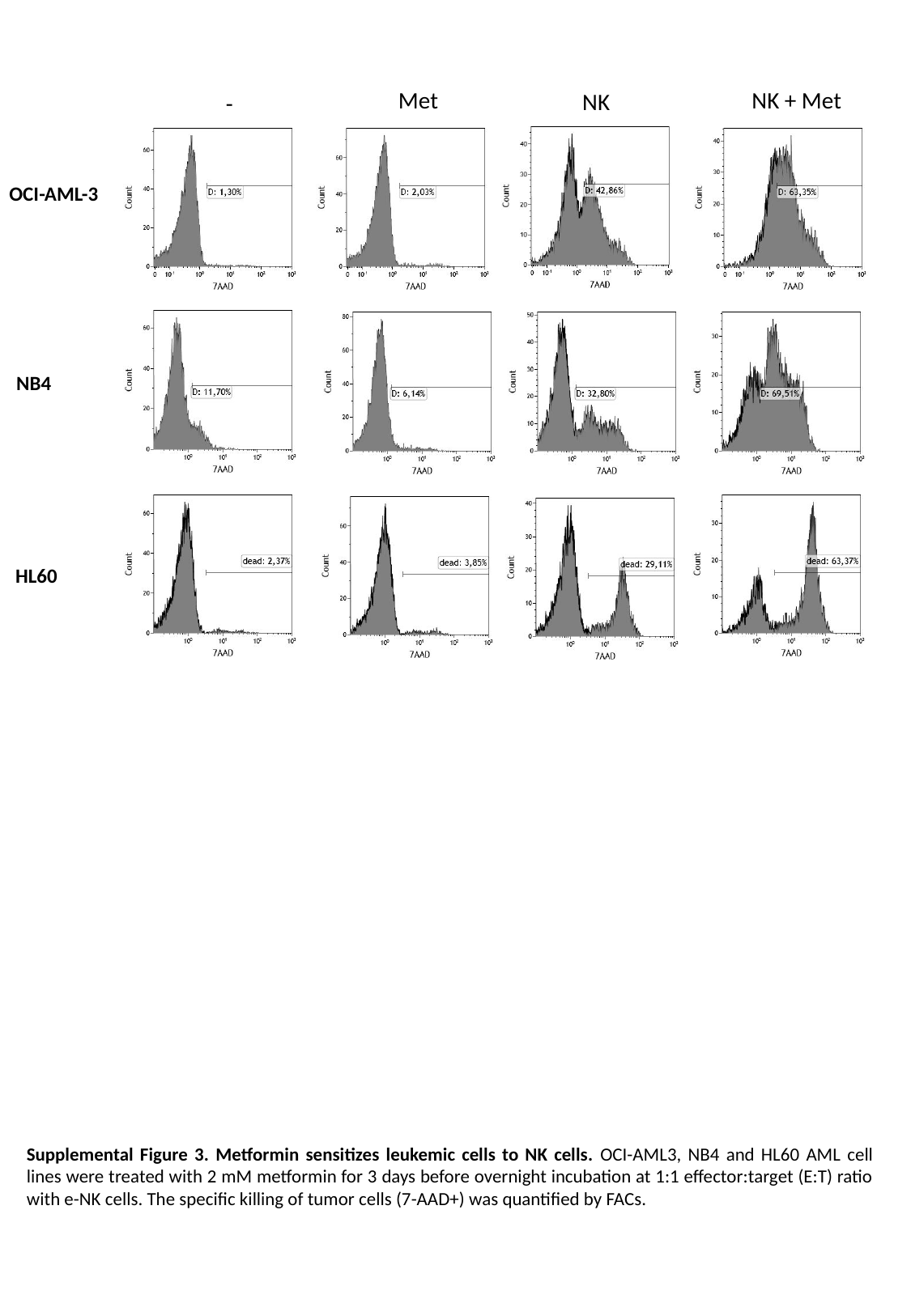

NK + Met
Met
NK
-
OCI-AML-3
NB4
HL60
Supplemental Figure 3. Metformin sensitizes leukemic cells to NK cells. OCI-AML3, NB4 and HL60 AML cell lines were treated with 2 mM metformin for 3 days before overnight incubation at 1:1 effector:target (E:T) ratio with e-NK cells. The specific killing of tumor cells (7-AAD+) was quantified by FACs.

## Slide 4
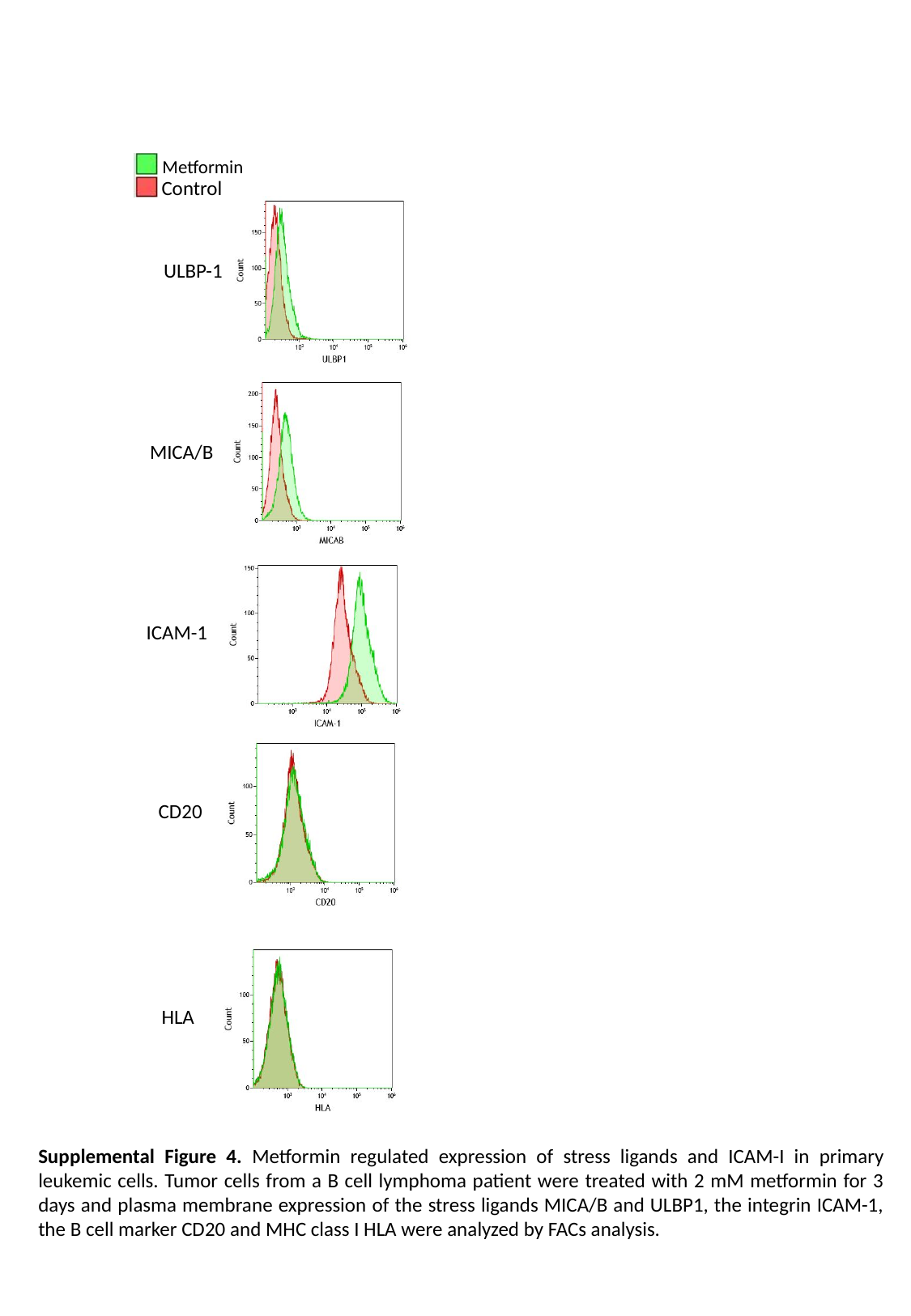

Metformin
Control
ULBP-1
MICA/B
ICAM-1
CD20
HLA
Supplemental Figure 4. Metformin regulated expression of stress ligands and ICAM-I in primary leukemic cells. Tumor cells from a B cell lymphoma patient were treated with 2 mM metformin for 3 days and plasma membrane expression of the stress ligands MICA/B and ULBP1, the integrin ICAM-1, the B cell marker CD20 and MHC class I HLA were analyzed by FACs analysis.

## Slide 5
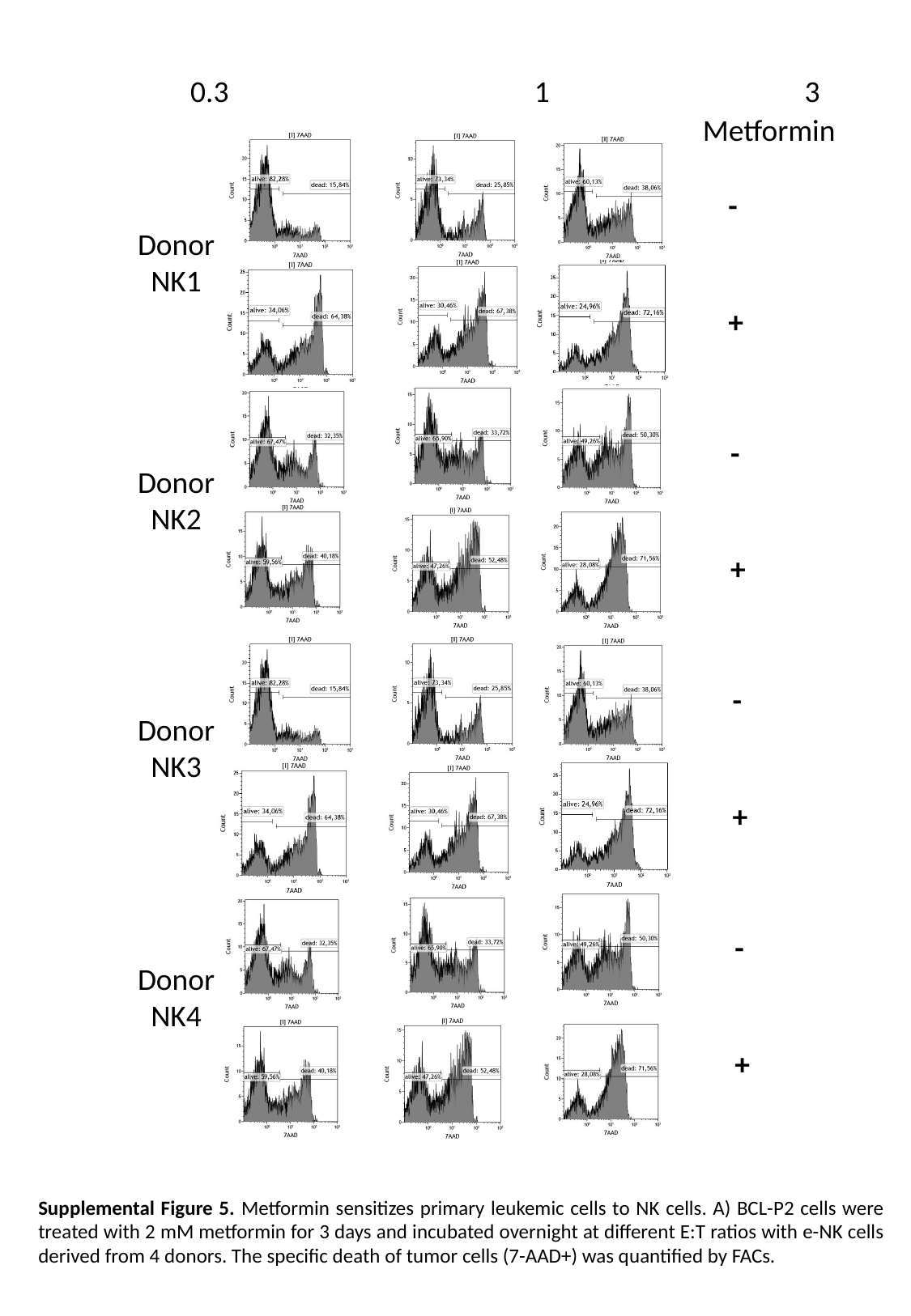

E:T		 0.3			1		 3
Metformin
-
Donor
NK1
+
-
Donor
NK2
+
-
Donor
NK3
+
-
Donor
NK4
+
Supplemental Figure 5. Metformin sensitizes primary leukemic cells to NK cells. A) BCL-P2 cells were treated with 2 mM metformin for 3 days and incubated overnight at different E:T ratios with e-NK cells derived from 4 donors. The specific death of tumor cells (7-AAD+) was quantified by FACs.
